# Comparison of clinical characteristics between neuromyelitis optica spectrum disorders with and without spinal cord atrophy

**DOI:** 10.1186/s12883-014-0246-4

**Published:** 2014-12-20

**Authors:** Yanqiang Wang, Aimin Wu, Xiaoyu Chen, Lei Zhang, Yinyao Lin, Shaoyang Sun, Wei Cai, Bingjun Zhang, Zhuang Kang, Wei Qiu, Xueqiang Hu, Zhengqi Lu

**Affiliations:** Multiple Sclerosis Center, Department of Neurology, The Third Affiliated Hospital of Sun Yat-sen University, No 600 Tianhe Road, Guangzhou, Guangdong 510630 China; Department of Neurology, The Fifth Affiliated Hospital of Sun Yat-sen University, Zhuhai, China; Department of Radiology, The Third Affiliated Hospital of Sun Yat-sen University, Guangzhou, China

**Keywords:** Neuromyelitis optica spectrum disorders, Spinal cord atrophy, Longitudinally extensive transverse myelitis, Magnetic resonance imaging

## Abstract

**Background:**

Spinal cord lesions is one of the predominant characteristics in patients with neuromyelitis optica spectrum disorders (NMOSD). Interestingly, mounting evidence indicates that spinal cord atrophy (SCA) is one of common clinical features in multiple sclerosis (MS) patients, and correlates closely with the neurological disability. However, Clinical studies related to the SCA aspects of NMOSD are still scarce.

**Methods:**

We retrospectively analyzed 185 patients with NMOSD, including 23 patients with SCA and 162 patients without SCA. Data were collected regarding clinical characteristics, laboratory tests, and magnetic resonance imaging findings.

**Results:**

12.4% of patients had SCA in NMOSD. Patients with SCA had a longer disease duration and higher EDSS at clinical onset and last visit. More importantly, SCA patients were more prone to reach disability milestones (EDSS ≥ 6.0). Bowel or bladder dysfunction, movement disorders, and sensory disturbances symptoms were more common in patients with SCA. ESR and CRP were significantly higher in patients with SCA than those without SCA. Patients with SCA were more frequently complicated with cervical cord lesions. However, the ARR, progression index, seropositive rate of NMO-IgG and OCB were similar in the two groups. Futhermore, LETM did not differ significantly between patients with SCA and without SCA in NMOSD patients.

**Conclusions:**

Patients with SCA might have longer disease duration, more severe clinical disability, and more frequently complicated with cervical spinal cord lesions. SCA might be predictive of the more severe neurologic dysfunction and worse prognosis in NMOSD. Inflammation contributes to the development of SCA in NMOSD.

## Background

Neuromyelitis optica spectrum disorders (NMOSD) is a group of inflammatory demyelinating disorders, mediated by pathogenic autoantibodies (NMO-IgG) against astrocyte aquaporin-4 (AQP4), the main water channel of the central nervous system (CNS) [1,2]. As it is well known that the spinal cord is one of the most frequently involved sites in NMOSD, especially longitudinally extensive spinal cord lesions have been observed in 72.4-100% of NMO [3-6], and are predominantly located in the cervical and upper thoracic region. Futhermore, the neurological function changes in spinal cord injury are considered as the clinical hallmark of the disease evolution [5,6].

The current studies indicate atrophy is a common pattern during the disease course and a potential marker of clinical disability in all subtypes of multiple sclerosis (MS) [7-9]. Spinal cord atrophy (SCA), particularly atrophy of cervical cord, is considered to contribute to accumulation of disability and clinical outcome [7,10]. SCA is expected to present in NMOSD. However, little attention has been paid to exploring the clinical features of SCA in NMOSD. Only a few sporadic studies have reported the frequent occurrence, locations of the SCA in exploring the features of spinal cord lesions with NMO patients [4,11-13]. Therefore, we investigated and compared the clinical, laboratory, and magnetic resonance imaging (MRI) characteristics between NMOSD with and without SCA.

## Methods

### Patients

We retrospectively reviewed the medical records of 185 patients with NMOSD (23 patients with SCA and 162 patients without SCA) who were hospitalized at the multiple sclerosis (MS) center of the Third affiliated hospital of Sun Yat-sen University between March 2008 and September 2013. All the patients were diagnosed according to the Wingerchuk 2006 and 2007 criteria [1,6,14,15]. And followed up in the outpatient once a month after discharge. Disability was assessed using the Expanded Disability Status Scale (EDSS), EDSS milestones (severe disability) at follow-up was defined as EDSS ≥ 6.0 [16,17]. Disease severity was evaluated by the progression index (Progression index = EDSS/disease duration) [18]. Relapses were defined as new or recurrent neurologic symptoms not associated with fever or infection that lasted ≥24 h and were accompanied by new neurologic signs found by the examining neurologist. Disease duration as measured in years since the onset of the first symptoms until last follow up, disease activity such as ARR (ARR = total number of relapses/disease duration) and total number of relapses [19,20]. Cerebrospinal fluid oligoclonal bands (OCBs), NMO-IgG, anti-nuclear antibodies (ANA), anti-SSA/Ro antibodies (SSA), anti-SSB/La antibodies (SSB), rheumatoid factor (RF), complement, ESR, CRP were tested at the time of the initial diagnosis, prior to corticosteroid treatment. All of the patients received high-dose corticosteroids pulses [(methylprednisolone 1 g, IV/d for 5d) for 2–3 courses, each treatment interval was three days] during the relapse period. And in remission period, all the patients are treated with oral small doses of prednisone (8–20 mg/d, oral) combined with azathioprine (50–100 mg/d). None of the patients had underwent therapeutic plasmapheresis. The patients were excluded who had anemia, hypoalbuminemia, infectious diseases, vascular diseases, metabolic disorders, and other inflammatory demyelinating diseases.

### Magnetic resonance imaging (MRI) scanning

A 1.5-T magnetic resonance imager (General Electric, Milwaukee, WI, USA) was used to perform the brain and spinal cord MRIs. Conventional MRI protocols were used in all patients: T1-weighted images (T1W) with and without gadolinium enhancement (GDE), T2-weighted images (T2W) and fluid attenuated inversion recovery (FLAIR). Brain MRI lesions were evaluated and defined according to the Paty criteria, and that described by Ito et al. [6,21-24]. Spinal cord atrophy were evaluated according to previous reports. Spinal cord atrophy were evaluated and measured according to previous reports of multiple sclerosis (MS). The whole spinal cord was defined by using bony landmarks (foramen magnum rostrally to the T12 vertebral body caudally). The spinal cord was segmented into cervical and thoracolumbar regions. and measured an upper cervical cord volume from C2 to C3 by first measuring the cross-sectional area of the cord at the level of the C2/C3 intervertebral disc. Cross-sectional area was assessed at the slice above and below the C2/C3 section. The mean volume of the three contiguous slices was calculated for each subject [8,9]. Longitudinally extensive transverse myelitis (LETM) is a spinal cord lesion that extends over 3 or more vertebral segments [6]. While shorter transverse myelitis (STM) lesions were defined as an area extending over less than three vertebral segments. All MRI scans were performed prior to use of corticosteroid, immunomodulatory or immunosuppressive treatment. An experienced neuroradiologist and a neurologist, both of whom were blinded to the diagnostic categorization and the patients’ clinical features. each analyzed all of the MRI scans. The final assessments were made by consensus.

### Statistical analysis

Statistical analysis was performed by SPSS version 13.0. Values of p = 0.05 were considered statistically significant. Categorical data were expressed as N, percentage, and analyzed with chi-square test. Continuous data with a normal distribution were expressed as the mean ± SD and further analyzed with an independent 2-sample student's t-test. Data that were not normally distributed were analyzed by the Wilcoxon Mann–Whitney U-test.

### Protocol approvals, registrations, and patient consent

The study was approved by the ethics committee of the Third Affiliated Hospital of Sun Yat-sen University and the informed consent was obtained from all subjects.

## Results

### Demographics and clinical characteristics of patients with and without SCA in NMOSD

**Figure 1:**
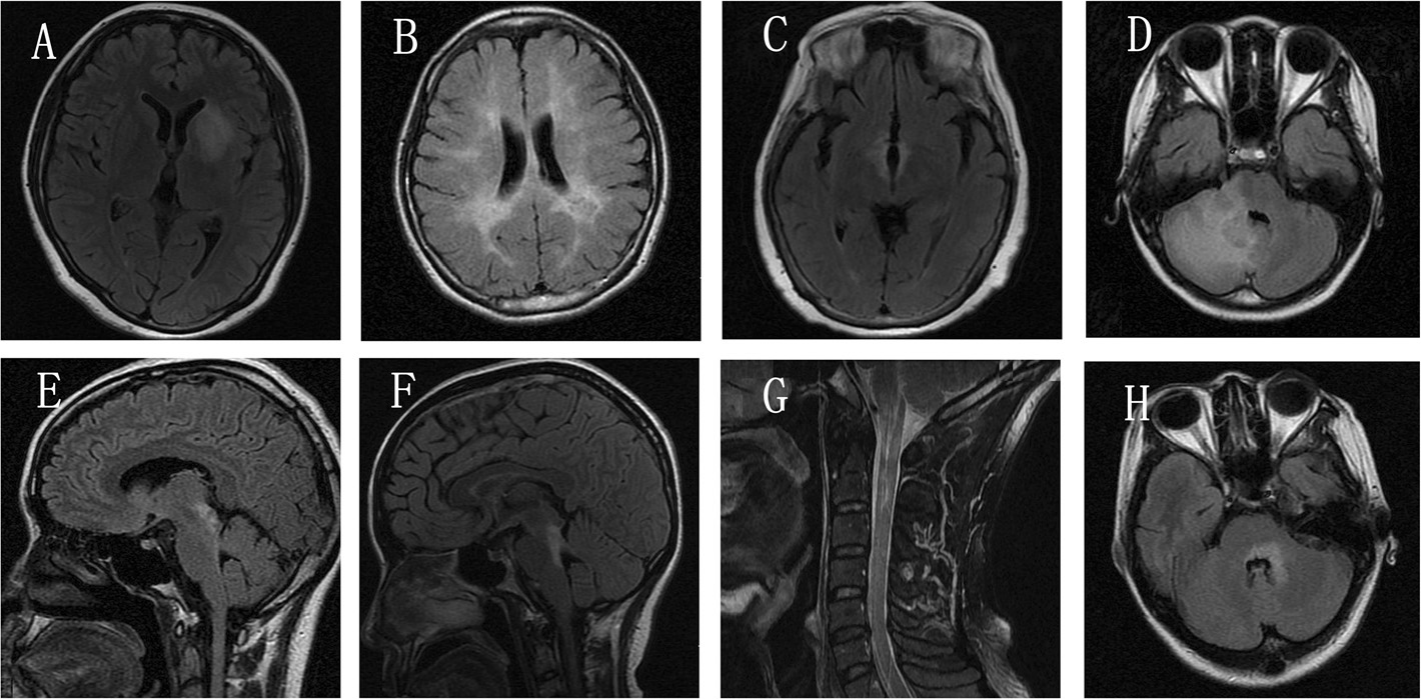
**Typical brain MRI lesions in neuromyelitis optica spectrum disorders (NMOSD) with spinal cord atrophy (SCA) lesions.** A-D, H: T2 FLAIR; E-G: T2 FRFSE, **A**. Lesions in basal ganglia, **B**. Lesions in periventricular area, **C**. Lesions in hypothalamic region, **D**. Lesions in cerebellar hemispheres, **E**. Lesions in midbrain aqueduct, **F**. Lesions in pons tegmental area, **G**. Lesions in the medulla oblongata (MO) and C1-2, **H**. Lesions in fourth periventricle area.

The clinical features of the study patients are shown in Table 1. The age, gender, age at onset were similar in NMOSD patients with and without SCA. No differences were found in ARR or progression index between these two groups. The patients with SCA had longer disease duration (p = 0.001), higher EDSS score (p = 0.001) than those without SCA. Importantly, had a more rapid rate of disease evolution and more severe physical disability from the clinical onset to last visit (EDSS ≥ 6.0) (p = 0.001 and p = 0.001, respectively). Compared to patients with the patients without SCA, the patients with SCA more frequently present with bowel or bladder dysfunction (p = 0.002), movement disorders (p = 0.001), and sensory disturbances (p = 0.027) (Figure 1).

**Table 1:** **Demographic and clinical characteristics of patients with or without SCA in NMOSD**

	**NMOSD with SCA (n = 23)**	**NMOSD without SCA (n = 162)**	**P**
Gender, F:M	19:4	133:29	0.952
Age, years	46.82 ± 11.17	42.05 ± 11.52	0.064
Age at onset, years	35.84 ± 13.99	38.49 ± 12.05	0.335
Disease duration, years	7.92 (0.5-30)	1.75 (0–39)	0.001**
SCA duration, years	4.58 (0.6-26)		
Annualized relapse rate	0.84 (0.07-3.23)	0.66 (0–12)	0.826
EDSS at clinical onset	6.02 ± 2.16	2.49 ± 2.01	0.001**
EDSS > 6 (at clinical onset)	14 (60.9%)	14 (8.6%)	0.001**
EDSS at last visit	5.37 ± 2.38	4.21 ± 1.80	0.006*
EDSS > 6 (at last visit)	11 (47.8%)	27 (28.9%)	0.001**
Reaching EDSS 6 from onset, n (%)	15 (65.62%)	30 (18.2%)	0.001**
Reaching EDSS 6 duration from onset, years	1 (0.08-6)	2.25 (0.08-23.08)	0.001**
Progression index	0.79 (0.14-12)	0.96 (0–90)	0.410
Clinical features, n (%)			
Headache	4 (17.4%)	21 (13%)	0.561
Dizziness	3 (13%)	23 (14.2%)	0.882
Nystagmus	4 (17.4%)	15 (9.3%)	0.229
IHN	6 (26.1%)	43 (26.5%)	0.963
Dysphagia or choking cough	3 (13%)	12 (7.4%)	0.354
Bowel or bladder dysfunction	16 (69.6%)	59 (36.4%)	0.002**
Visual impairment	19 (82.6%)	131 (80.9%)	0.842
Movement disorders	22 (95.7%)	82 (50.6%)	0.001**
Sensory disturbances	21 (91.3%)	112 (69.1%)	0.027*
Neuropathic pain	8 (34.8%)	54 (33.3%)	0.890
Early standard corticosteroid therapy (<7 days of onset)	9 (39.1%)	128 (70.01%)	0.0539

NMOSD = neuromyelitis optica spectrum disorders; SCA = spinal cord atrophy; ARR = Annualized relapse rate; EDSS = Expanded Disability Status Scale; IHN = intractable hiccup and nausea; SCA duration = Duration between at the onset of NMOSD and at the appearance of SCA; *P < 0.05; **P < 0.01; P values also reflect comparison of percentages in clinical features.

### Laboratory tests of patients with and without SCA in NMOSD

The data of laboratory tests were summarized in Table 2. There were no significant differences in CSF, immunological indexes and other antibodies between patients with and without SCA. However, the level of the ESR and CRP were much higher in patients with SCA than those without SCA (19 vs 12, p = 0.045; 2.45 vs 0.9, p = 0.001, respectively). Although significant differences between the patients with and without SCA existed in the positive ratio of OCB and NMO-IgG. But, no remarkable change was observed in the two groups (Table 3).

**Table 2:** **Clinical characterisation and MRI follow-up of NMOSD patients with SCA (n = 23)**

**Clinical manifestations**		**NMO-IgG positive (n = 16)**	**NMO-IgG negative (n = 7)**
F/M		13/3	6/1
Duration of disease	0-4 y	6	2
	5-9 y	5	2
	10- y	5	3
Brain lesion(n)			
Brain lobes		8	7
Basal ganglia		3	2
Hypothalamic and thalamic		4	2
Mesencephalon		1	1
Pons		5	3
Medulla oblongata		4	2
Peri-ventricle and peri-aqueduct		4	3
Cerebellum		3	0
Spinal cord lesions(n)			
LETM		21	5
STM		8	4
Focal atrophy of SC		14	5
General atrophy of SC		6	3
Atrophy of STM		13	3
Atrophy of LETM		9	4

NMOSD = neuromyelitis optica spectrum disorders; SCA = spinal cord atrophy; NMO-IgG = anti-AQP4IgG autoantibodies; LETM = longitudinally extensive transverse myelitis; STM = shorter transverse myelitis; SC = Spinal cord.

**Table 3:** **Biochemical values of patients with or without SCA in NMOSD**

		**NMOSD with SCA (n = 23)**	**NMOSD without SCA (n = 162)**	**P**
CSF Index	WBCs (10^6^)	7 (2–20)	4 (0–70)	0.807
	Protein (0.15-0.4 mg/ml)	0.23 (0.12-0.68)	0.24 (0.06-0.95)	0.395
	Glucose (2.5-3.9 mg/ml)	3.5 (2.52-6.64)	3.28 (1.81-6.64)	0.151
	Chloride (121.0-129.0 mg/ml)	127.6 (100.9-131.6)	126.95 (100.9-138.5)	0.134
	OCB (+), n (%)	0/10 (0%)	11/75 (14.7%)	0.174
Serums Index	CRP (0–6 mg/l)	2.45 (0–62.5)	0.9 (0–20.5)	0.001**
	ESR (0–20 mm/H)	19 (6–91)	12 (1–91)	0.045*
	NMO-IgG (+), n (%)	16/23 (69.6%)	44/58 (75.9%)	0.155
	ANA (+), n (%)	7/13 (53.8%)	40/91 (44%)	0.503
	SSA (+), n (%)	2/15 (13.3%)	16/89 (18%)	0.660
	SSB (+), n (%)	2/15 (13.3%)	8/89 (9%)	0.597
	RF (+), n (%)	5/14 (30.8%)	7/51 (13.7%)	0.146
	IgG (8–16 g/L)	12.58 (7.56-30.36)	11.9 (1.33-33.22)	0.483
	IgA (0.7-3.3 g/L)	1.56 (0.19-4.59)	1.57 (0.7-4.34)	0.894
	IgM (0.5-2.2 g/L)	1.03 (0.05-1.96)	1.19 (0.45-6.54)	0.128
	C3 (0.8-1.6 g/l)	0.99 (0.13-1.66)	1.06 (0.51-2.11)	0.529
	C4 (0.1-0.4 g/l)	0.22 (0.11-0.32)	0.2 (0.04-1.27)	0.719
	CH50 (23–46 U/ml )	37 (16–71)	47 (10–65)	0.103

NMOSD = neuromyelitis optica spectrum disorders; SCA = spinal cord atrophy; CSF = cerebrospinal fluid; OCB = oligoclonal banding; CRP = C-reactive protein; ESR = erythrocyte sedimentation rate; NMO-IgG = anti-AQP4IgG autoantibodies; ANA = antinuclear antibodies; SSA = anti-SSA/Ro antibodies; SSB = anti-SSB/La antibodies; RF = rheumatoid factor; *P < 0.05; **P < 0.01.

### Characteristics of brain and spinal cord lesions on MRI of patients with and without SCA in NMOSD

The characteristics of brain and spinal cord lesions on MRI were summarized in Table 3. Although, extensive brain lesions were frequently involved in patients with SCA, including brain lobes, basal ganglia, hypothalamic and thalamic, medulla oblongata, pons, diencephalon, lateral ventricle, third ventricle and aqueduct, and cerebellum. However, there was no statistically significant difference between the patients with and without SCA (Figure 1, Tables 2 and 4).

**Table 4:** **Comparative brain and spinal cord lesions on MRI of patients with or without SCA in NMOSD**

	**NMOSD with SCA (n = 23)**	**NMOSD without SCA (n = 162)**	**P**
Brain lesions, n (%)			
Brain lobes	15 (65.2%)	71 (46.5%)	0.054
Basal ganglia	5 (21.7%)	26 (16%)	0.494
Hypothalamic and thalamic	6 (26.1%)	19 (11.7%)	0.059
Medulla oblongata	8 (34.8%)	31 (19.1%)	0.085
Pons	8 (34.8%)	32 (19.8%)	0.101
Mesencephalon	2 (8.7%)	6 (4.3%)	0.271
Peri-ventricleand peri-aqueduct	7 (30.4%)	35 (21.6%)	0.344
Cerebellum	3 (13%)	8 (4.9%)	0.124
Spinal cord lesions, n (%)			
Cervical cord	20 (87%)	79 (48.4%)	0.001**
Segments lesions	4.74 ± 1.91	4.65 ± 1.87	0.849
LETM	13 (56.5%)	62 (59%)	0.842
STM	10(43.5%)	32 (30.5%)	0.229
Thoracic cord	3 (13%)	62 (38.3%)	0.018^*^
Segments lesions	6.00 ± 3.37	5.53 ± 2.93	0.549
LETM	18 (73.9%)	67 (65.7%)	0.448
STM	6 (26.1%)	26 (24.8%)	0.894
Cervical and thoracic cord	17 (73.9%)	45 (27.8%)	0.001^**^

MRI = magnetic resonance imaging; SCA = spinal cord atrophy; NMOSD = neuromyelitis optica spectrum disorders; LETM = longitudinally extensive transverse myelitis; STM = shorter transverse myelitis; *P < 0.05; **P < 0.01.

**Figure 2:**
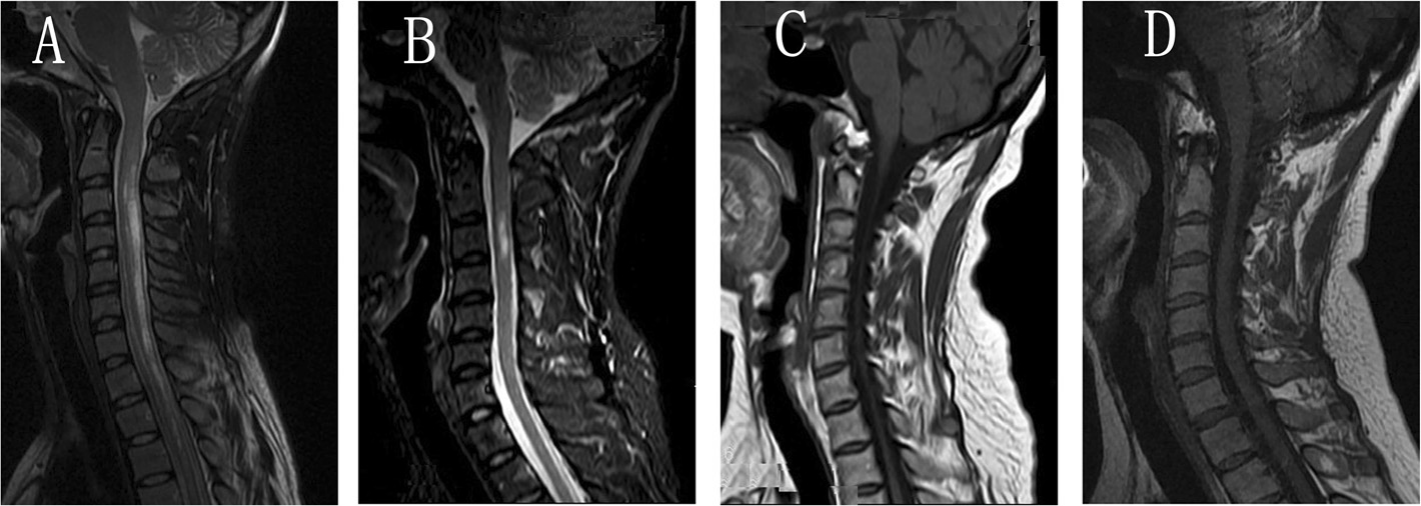
**Typical transverse myelitis (TM) and atrophy spinal cord MRI lesions in neuromyelitis optica spectrum disorders (NMOSD).** Representative MRI of four NMOSDs patients with SCA. Spinal cord MRI: sagittal T2 FRFSE **(A and** **B)**, T1WI **(C and** **D)**. **A**. MRI showing LETM of spinal (C2-T3)cord. **B**. MRI showing STM of cervical(C3)cord. **C**. MRI showing atrophy of LETM(C1- T2). **D**. MRI showing atrophy of STM(C2-C3).

The spinal cord lesions were predominantly located in cervical and thoracic cord in both groups. But the ratio of cervical cord involvement was significantly higher in the patients with SCA than those without SCA (p = 0.001). Furthermore, cervical and thoracic cord lesions were more frequently found in patients with SCA than that in the patients without SCA (p = 0.001). Interestingly, no statistically significant difference was noted between the two groups for the length of lesions in cervical and thoracic cord measured by vertebral segments (p = 0.849, p = 0.549, respectively). Moreover, the ratio of longitudinally extensive transverse myelitis of cervical and thoracic cord were similar in the two groups (p = 0.842, p = 0.448, respectively) (Figure 2, Tables 2 and 4).

## Discussion

Although the spinal cord lesion is widely recognized as one of the predominant characteristics in NMOSD. However, the SCA has only been sparsely studied, and only focused on the locations, incidences and magnetic resonance imaging markers of SCA in NMOSD patients [4,11]. To the best of our knowledge, this is the first study to investigate the clinical characteristics of SCA in Chinese NMOSD patients. In the present study, we confirmed that the SCA patients had more frequently cervical spinal cord lesions, more severe clinical disability, and longer disease duration. More importantly, our findings suggest inflammation play the important role in the development of SCA in NMOSD.

Previous reports revealed that the frequent occurrence of SCA in NMO patients was 52.2% or 57% [4,11], respectively. However, in our study, the prevalence of SCA was 14.2% in adult Chinese patients with NMOSD. and SCA mainly located in the cervical spinal cord. Factors influencing variation may include differences in disease susceptibility, diagnostic criteria, sample size. The spinal cord, especially the cervical cord, has been identified as containing important ascending and descending pathways related to locomotion and sensation. Therefore, any degree of SCA may be associated with the presence of sensory, limb movements, or urinary symptoms [7,10]. In our SCA patients, the clinical relevance of SCA in NMOSD was supported by the difference which we found EDSS score, particularly reaching disability milestones at clinical onset and last visit (EDSS ≥ 6.0); the time to reach EDSS 6.0 from onset; the more frequency and severity of clinical presentation including bowel or bladder dysfunction, movement disorders and sensory disturbances between the two group patients. Therefore, the neurological impairment to reach EDSS ≥ 6.0; residual EDSS score; a greater number of functional systems involved at onset as well as higher residual deficits in pyramidal, visual, sphincteric and sensory system, may be the predictors of favorable SCA. Besides, disease duration was also one of the vital factors contributing to SCA, this suggesting a cumulative effect with time of the pathologic processes leading to progressive and irreversible tissue lesion in the cord [7,10].

More recent studies suggest a potential link inflammation with disease activity and clinical disability of NMO and MS [25-27]. The C-reactive protein(CRP) is an acute phase protein and a component of the innate immune system. Current studies indicate CRP was produced by astrocytes [28]. it could assess the degree of inflammation, correlate with disease activity and clinical relapse in MS and NMO [29-31]. In our study, we found CRP and ESR in NMOSD patients with SCA were higher than those without SCA. These suggest that the SCA patients may have more severe systemic inflammatory reactions, and disease activity. Moreover, dysfunction of astrocytes occur early in NMO pathophysiological process, and trigger demyelination, myelin loss, neuron death, and promote SCA formation in NMOSD. Meanwhile, the innate immune system may also play a critical role in the initiation and progression of SCA by mediating the demyelination of neuronal axons, and initiating a cascade of immuno-inflammatory reactions. Although some studies reported hypercomplementemia (serum C4, CH50 and CSF - C5a, sC5b-9) was significantly higher in NMO patients, especially in anti-AQP4 antibody positive patients [32-34]. However, no significant difference was found in complements between the two groups. And few data showed the relationships between complements and SCA in NMOSD. So further work is needed to determine whether complements promotes SCA formation. AQP4 antibodies act as a radical initiator to induce the NMOSD lesion, and consistently play an important role in the pathological process by monitoring inflammatory tissue injury, demyelination, necrosis and axonal damage [35-37]. However, in the present study, there were no differences in the seropositivity of NMO-IgG between patients with and without SCA. So NMO-IgG might not be associated with SCA. But, due to technical reasons, we did not test the titer of NMO-IgG which were thought to be related to the disease progress and exacerbation [6,38]. However, any suggestion of a positive link between the NMO-IgG titers and SCA must be prompt further research using the larger, prospective study design. Besides, although NMO-IgG and systemic inflammatory reactions were closely associated

with astrocytes and involved in the pathogenesis of NMOSD [28,39]. However, our results suggest that they may be a two stage of the SCA disease course, or have potential mechanisms for "cross-talk" between the NMO-IgG and systemic inflammatory reactions.

Clearly, this study is not without limitations. Because of the technical conditions, the titers of some autoantibodies and NMO-IgG could not be tested; because only a limited number of patients with SCA, we were not able to make a comprehensive analysis on different anti-AQP4 antibody positive/negative of SCA.

## Conclusions

The patients diagnosed as NMOSD with SCA have more severe disability, and longer disease duration. In the early phases of the disease, the SCA, specially concomitant cervical spinal cord lesions, may be an important indicator of disease severity, and a limited form event of NMOSD. Systemic inflammation occurs rapidly after the onset of SCA, and is considered as one of the important components of neurodegenerative processes in SCA. Thus, early and effective inhibition of systemic microinflammation is critical to prevent or minimize the development of the SCA in NMOSD. Measurements of SCA might help to predict disease severity and evaluate the therapeutic effects of NMOSD.

## Abbreviation

NMOSD: Neuromyelitis optica spectrum disorders
SCA: Spinal cord atrophy
ARR: Annualized relapse rate
EDSS: Expanded disability status scale
PI: Progression index
IHN: Intractable hiccup and nausea
SCA duration: Duration between at the onset of NMOSD and at the appearance of SCA
NMO-IgG: Anti-AQP4IgG autoantibodies
LETM: Longitudinally extensive transverse myelitis
STM: Shorter transverse myelitis
SC: Spinal cord
CSF: Cerebrospinal fluid
OCB: Oligoclonal banding
CRP: C-reactive protein
ESR: Erythrocyte sedimentation rate
ANA: Antinuclear antibodies
SSA: Anti-SSA/Ro antibodies
SSB: Anti-SSB/La antibodies
RF: Rheumatoid factor
MRI: Magnetic resonance imaging
